# Cultivation of Drought-Tolerant and Insect-Resistant Rice Affects Soil Bacterial, but Not Fungal, Abundances and Community Structures

**DOI:** 10.3389/fmicb.2018.01390

**Published:** 2018-06-29

**Authors:** Peng Li, Shuifeng Ye, Hua Liu, Aihu Pan, Feng Ming, Xueming Tang

**Affiliations:** ^1^The Biotechnology Research Institute, Shanghai Academy of Agricultural Sciences, Shanghai, China; ^2^State Key Laboratory of Genetic Engineering, Institute of Genetics–Institute of Plant Biology, School of Life Sciences, Fudan University, Shanghai, China; ^3^Shanghai Agrobiological Gene Center, Shanghai, China

**Keywords:** drought-tolerant and insect-resistant rice, Illumina MiSeq sequencing, Bt proteins, bacterial community composition, fungal community composition

## Abstract

The impacts of rice varieties with stacked drought tolerance and insect resistance on soil microbiomes are poorly understood. Hence, the objective of this study was to investigate the effects resulting from the cultivation of the drought-tolerant and insect-resistant rice cultivar, Hanhui3T, on soil physical–chemical properties, and bacterial and fungal community composition. Soil samples of Hanhui3T and conventional rice varieties (Hanhui3 and Zhonghua11) were collected in triplicate at the booting stage, and bacterial and fungal population sizes and community structures were assessed using qPCR and Illumina MiSeq sequencing, respectively. The Bt protein concentration of Hanhui3T was significantly higher than that of Hanhui3 and Zhonghua11, while the pH of Hanhui3T was significantly lower. Bacterial population sizes and community composition were significantly different between Hanhui3T and Hanhui3 (or Zhonghua11), while no similar effects were observed for fungal communities. These differences suggest that the effect of Hanhui3T cultivation on bacterial community composition is stronger than the effect on fungal communities. Moreover, bacterial abundance was positively correlated to soil pH, while bacterial community structure variations were mainly driven by soil pH and Bt protein concentration differences. In conclusion, the abundances and structure of bacterial communities were altered in rhizosphere with Hanhui3T cultivation that changed soil pH and Bt protein concentrations, while fungal communities displayed no such responsiveness.

## Introduction

The rhizosphere represents a hot spot for microbial activity and constitutes one of the most complex ecosystems on earth, with numerous complex interactions with neighboring plants and microbes (Mendes et al., 2013). Plant root exudates initiate and modulate the relationships between roots and soil microbes, whereby the quantity and quality of root exudates are determined by plant genotypes (Badri and Vivanco, 2009). Consequently, these relationships are often specific, indicating that changes in plant genotypes impact the association of specific microbial groups with plants, and thus altering the abundance and composition of the rhizosphere microbiome. The alteration of plant genotypes by genetic modification have a potential bearing on the organisms that are targeted by the modification, in addition to other potential (collateral) effects resulting from the modification (Cotta et al., 2014). There has been a remarkable decline in crop planting area and food production in areas with continued deterioration of drought conditions brought about by climate change (Clive, 2016). Thus, the development of biotech crops with multiple favorable traits, such as drought and insect resistance, has increased dramatically, and rapid approval of these crops is needed.

A drought-tolerant and insect-resistant rice cultivar, Hanhui3T, has been developed by the Shanghai Agrobiological Gene Center of China. Field experiments have shown that Hanhui3T displays significant resistance to rice leaf roller, and apparently combines drought-tolerant traits (Ye et al., 2012). Importantly, the potential environmental effects of newly developed cultivars must be evaluated through environmental risk assessment before the cultivation of transgenic plants for commercial purposes (National Research Council, 2002). Environmental risk assessments are based on precautionary principles and they are practiced on a case-by-case basis by taking into consideration the nature of the plant, trait and the potential receiving environment (Khan et al., 2017). One of the major potential environmental risks associated with the use of drought- and insect-resistant rice varieties is the effect on soil and the inhabiting non-target organisms, including bacteria and fungi. The bacterial and fungal communities inhabiting the rhizosphere, that is the soil influenced by root metabolites, are influenced by soil types, plant species, local climates, and plant growth stages (Buée et al., 2009; Dohrmann et al., 2013). Sohn et al. (2016) suggest that the effects of drought-tolerant rice cultivation on soil microorganisms are not significant using plate counts and pyrosequencing. However, studies of the impact of transgenic rice with different genetic traits on rhizosphere microbial communities have revealed inconsistent results (Turrini et al., 2015). Moreover, considering the Bt protein that is released in root exudates from transgenic Bt plants (Saxena et al., 1999) and variation in drought stress-response gene expression, we hypothesized that drought- and insect-resistant rice varieties are likely to produce unintended effects on soil bacterial and the fungal communities. In addition, it is clear that factors, such as soil physical–chemical properties and plant genotypes, greatly influence the corresponding rhizosphere microbial community compositions (Khan et al., 2017). The extent to which these factors, including soil Bt protein exposure, influence and contribute to indigenous microbial community is not yet fully understood. Kennedy et al. (2004, 2005) hypothesized that bacterial communities are more sensitive to changes in environmental factors than fungal communities. However, additional studies are needed to evaluate whether bacterial and fungal community compositions differentially respond to the cultivation of drought and insect-resistant rice.

The objective of the present study was to characterize the effects of drought-tolerant and insect-resistant rice cultivation on the abundance and structure of soil bacterial and fungal communities. Bacterial and fungal population sizes were assessed with quantitative PCR (qPCR) of bacterial 16S rRNA genes and fungal internal transcribed spacer (ITS) genes, respectively. Changes in community structures (via 16S and 18S rRNA gene composition) between drought-tolerant and insect-resistant rice and conventional rice varieties were then assessed using Illumina MiSeq sequencing. Lastly, the relationships between soil bacterial and fungal community structures and soil physicochemical factors, including Bt protein exposure, were analyzed with redundancy analysis (RDA).

## Materials and Methods

### Plants and Field Design

Hanhui3T is a rice hybrid that produces the insecticidal delta endotoxin protein Cry1Ab/Ac, which confers resistant to pests and combines drought-tolerance traits. The Hanhui3T is bred by introducing the *Cry1Ab/Ac* gene into the rice cultivar Hanhui3 by agrobacterium-mediated genetic transformation (Ye et al., 2012). A variety of the hybrid, Hanhui3, was included in this study that is a near isogenic parental counterpart to Hanhui3T, in addition to the conventional variety, Zhonghua11. Seeds of Hanhui3T, Hanhui3, and Zhonghua11 were obtained from the Shanghai Agrobiological Gene Center (Shanghai, China). Plants of the three rice varieties were grown in the same chambers within a glasshouse at the Shanghai Academy of Agricultural Sciences. The experiment consisted of a randomized block design with nine replicate chambers, where each chamber had a volume of 100 cm × 100 cm × 30 cm. Each chamber contained plants of all three varieties (36 plants of each), which were planted with a distance of 15 cm between plants. The soil was classified as Fluvio marine blue purple clay and contained 13.3 g/kg of organic matter, 1.2 g/kg of total nitrogen, 90.5 mg/kg available N, and a pH of 7.4 (soil:water ratio 1:5). The seedlings were planted on May 30, 2016 and rice rhizospheres were sampled at the booting stage on August 25, 2016. After weeds and litter were removed from the soil surface, two types of soil samples were harvested. Bulk soil was excised from at least 4 mm away from any roots, and soils within 2 mm of the root surface were considered rhizosphere soils (DeAngelis et al., 2009). Plants were gently removed from soils and rhizospheres were collected by gently shaking roots to dislodge small adhering soil clumps. To ensure representativeness of samples, each sample was a composite from three chambers with the same rice variety. The samples were placed on ice in a cooler and transported to the laboratory on the same day. Soils were passed through a 2.0-mm sieve, and then a portion of the soil sample (collected in triplicate for each rice variety) was stored at -80°C before DNA extraction, while the remainder was stored at 4°C prior to the analysis of soil properties.

### Analysis of Soil Physicochemical Properties and Soil Microbial DNA Extraction

Soil Cry proteins were quantified by enzyme-linked immunosorbent assays as described by Dohrmann et al. (2013). Briefly, 0.5 g of soil sample was added to a 2-mL microcentrifuge tube. Next, 1.0 mL extraction buffer (0.1 M Na_2_CO_3_, 0.1 M NaHCO_3_, 5.0 mM EDTA, 50 mM Na_4_P_2_O_7_.10H_2_O, 0.1%Triton X-100, pH10) was added to fill the tube and the sample was mixed for 30 min at room temperature, followed by centrifugation for 5 min at 4°C at 12,000 ×*g*. The supernatant was collected to measure Bt protein content using a commercial ELISA kit (QuantiPlate^TM^ Kit for Cry1Ab/Cry1Ac, EnviroLogix Inc., Portland, ME, United States) according to the manufacturer’s protocol. Soil pH was determined by preparing a suspension of air-dried soil sample in water at a ratio of 1:5 (w/v), and pH was measured using a digital pH meter. Organic matter was determined using the potassium dichromate oxidation method (Lu, 2000), total nitrogen with the Kjeldahl procedure (Kirk, 1950), and available nitrogen (AN) by the hot alkaline permanganate method (Subbiah and Asija, 1956). Soil microbial DNA was extracted from the soil samples collected in triplicates as previously described (Li et al., 2014). Total genomic DNA was extracted from 0.5 g of sample using the FastDNA SPIN Kit for soil (MP Biomedicals, LLC, Solon, OH, United States). Purified DNA was stored at -80°C for the analyses of qPCR and Illumina MiSeq sequencing.

### Quantification of Bacterial and Fungal Community Abundances

Population sizes of soil bacterial and fungal communities were determined by qPCR assays according to methods described previously (Li et al., 2018), using the universal bacterial 16S rRNA gene primers 1369F (5′-CGG TGA ATA CGT TCY CGG-3′) and 1492R (5′-GGW TAC CTT GTT ACG ACT T-3′) (Suzuki et al., 2000), and fungal ITS genes primers NSI1 (5′-GAT TGA ATG GCT WAG TGA GG-3′) and 58A2R (5′-CTG CGT TCT TCA TCG AT-3′) (Mitchell and Zuccaro, 2006). The average bacterial and fungal qPCR efficiencies were 95.91 and 102.45%, respectively, with standard curve *R*^2^ values of 0.998 and 0.997, respectively.

### Illumina MiSeq Sequencing of Bacterial 16S rRNA and Fungal 18S rRNA Genes

Twelve independent samples of the rhizosphere from the three rice varieties Hanhui3T, Hanhui3, and Zhonghua11, and bulk soil as a control (with three replicates each) were selected for Illumina MiSeq sequencing. Bacterial 16S rRNA gene fragments were amplified with the primers 515F (5′-GTG CCA GCM GCC GCG G-3′)/907R (5′-CCG TCA ATT CMT TTR AGT TT-3′) and fungal 18S rRNA gene fragments were amplified with the primers SSU0817F (5′-TTA GCA TGG AAT AAT RRA ATA GGA-3′)/SSU1196R (5′-TCT GGA CCT GGT GAG TTT CC-3′) using a thermocycler PCR system (GeneAmp 9700, ABI, United States). PCR reactions were performed in triplicate in 20 μl reaction mixtures containing 4 μl of 5× FastPfu buffer, 2 μl of 2.5 mM dNTPs, 0.8 μl of each primer (5 μM), 0.2 μl of BSA, 0.4 μl of FastPfu polymerase, 10 ng of template DNA, and deionized, distilled water added up to a total volume of 20 μl. Bacterial PCRs comprised the following steps: 3 min of denaturation at 95°C, followed by 27 cycles of 95°C for 30 s, annealing at 55°C for 30 s, elongation at 72°C for 45 s, and a final extension at 72°C for 10 min. Fungal PCRs were conducted using the following program: 3 min of denaturation at 95°C, followed by 35 cycles of 95°C for 30 s, annealing at 53°C for 30 s, elongation at 72°C for 45 s, and a final extension at 72°C for 10 min. The resultant PCR products were visualized on a 2% agarose gel, and then extracted and purified using an AxyPrep DNA Gel Extraction Kit (Axygen Biosciences, Union City, CA, United States). PCR products were quantified using QuantiFluor^TM^-ST (Promega, United States) according to the manufacturer’s protocol. Equimolar amounts of the PCR products were pooled and prepared for sequencing on the Illumina MiSeq platform at Majorbio Bio-Pharm Technology Co., Ltd., Shanghai, China.

Raw sequence data were processed and analyzed using QIIME Pipeline version 1.8.0^1^ (Caporaso et al., 2010). Briefly, low-quality sequences that were shorter than 200 bp and had an average quality score of less than 20 were removed prior to analysis. Chimeric sequences were detected and removed using the UCHIME algorithm (Edgar et al., 2011). The remaining high-quality sequences were clustered into operational taxonomic units (OTUs) at the 97% sequence similarity level using the program: Cluster Database at High Identity with Tolerance (CD-HIT) (Li and Godzik, 2006). The taxonomy of each OTU was assessed using the RDP Classifier algorithm^2^ against the SILVA (128/16S Bacteria) 16S rRNA and SILVA (128/18S Eukaryota) 18S rRNA gene databases and a confidence threshold of 70%. All of the bacterial 16S rRNA and fungal 18S rRNA gene sequence data analyzed in this study have been deposited in the GenBank short-read archive SRA accessions: SRP130072 and SRP130195, respectively. In order to compare relative differences among samples, a subset of 11,620 and 21,723 bacterial and fungal sequences (the lowest value for a sample in each dataset), respectively, were randomly selected from each sample.

### Statistical Analyses

Beta-diversity estimates (Bray–Curtis and Hellinger distances) were calculated using the QIIME pipeline (Caporaso et al., 2010). To estimate alpha-diversity, the Shannon–Wiener index *H′*, Simpson index *D*, Richness *R*, and Chao1 indices were calculated and rarefaction curves were generated using the mothur software package (v 1.35.1) (Schloss et al., 2009). Hierarchical cluster analysis of samples based on Hellinger distances was performed using the R software package^3^. The statistical significance of differences in soil properties, 16S rRNA and ITS gene copies, and bacterial and fungal alpha-diversity among samples were examined by one-way analysis of variance (ANOVA) using SPSS 19.0 (SPSS Institute, Inc., 2010), and the shortest significant range (SSR) test was employed for multiple *post hoc* comparisons. In addition, the relationships between soil properties and 16S rRNA and ITS gene copies were tested by linear regression analyses using SPSS 19.0. Significant differences were considered as *P* < 0.05. An analysis of similarities (ANOSIM) was performed using QIIME 1.8.0 software (Caporaso et al., 2010) to determine whether different soil samples had significantly different microbial communities. RDA was conducted to determine the environmental variables that were most related to bacterial and fungal community compositional differences, and the results were used to construct a soil property matrix for variation partitioning analysis in R (v.2.8.1) using a Mantel test with the Pearson correlation method and 1,000 permutations through the “vegan” package (v.1.15-1) (R Core Team, 2006).

## Results

### Soil Properties

The concentrations of soil Bt protein, organic matter, total N, and available N, in addition to pH are shown in **Table 1**. Cultivation of drought-tolerant and insect-resistant rice resulted in increased soil Bt protein content. The Bt protein content of the three rice varieties’ rhizosphere and bulk soils were between 49.34 and 75.39 pg/g soil. The Bt protein content in the Hanhui3T rhizosphere was significantly higher than that in the other samples. In addition, the soil pH of Hanhui3T was significantly lower than that of Hanhui3 and Zhonghua11, but no significant difference was noted between Hanhui3T and bulk soils. No differences in organic matter, total N, or available N contents were found among all of the samples.

**Table 1:** Physical–chemical characteristics of the soil samples used in this study^a^.

Rice Sample	pH (1:5 H_2_O)	Bt protein (pg/g)	Organic matter (g/kg)	Total N (g/kg)	Available N (mg/kg)
Hanhui3T	7.46 ± 0.01^b^	75.39 ± 9.95^a^	14.03 ± 1.45^a^	1.35 ± 0.04^a^	90.90 ± 2.04^a^
Hanhui3	7.53 ± 0.05^a^	49.34 ± 2.41^b^	14.46 ± 0.87^a^	1.35 ± 0.08^a^	91.84 ± 1.39^a^
Zhonghua11	7.55 ± 0.06^a^	53.00 ± 7.00^b^	14.73 ± 0.55^a^	1.39 ± 0.06^a^	93.40 ± 0.95^a^
BS	7.41 ± 0.06^b^	49.67 ± 4.73^b^	13.90 ± 0.56^a^	1.28 ± 0.04^a^	91.12 ± 1.90^a^

^a^*Values represent the averages of three repetitions and the standard deviations. Different lowercase letters in the same column indicate a statistically significant difference at 0.05 level. BS indicates bulk soil control*.

### qPCR Estimates of the Total Bacterial and Fungal Population Sizes

**FIGURE 1:**
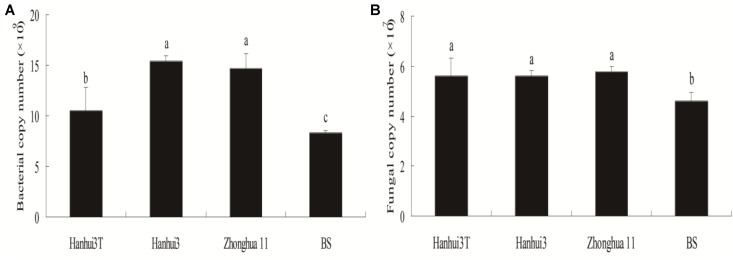
Copy numbers of the 16S rRNA **(A)** and ITS **(B)** genes from the rhizosphere microbial DNA extracts (Mean ± Standard Deviation) as determined by qPCR. BS indicates bulk soil control.

**FIGURE 2:**
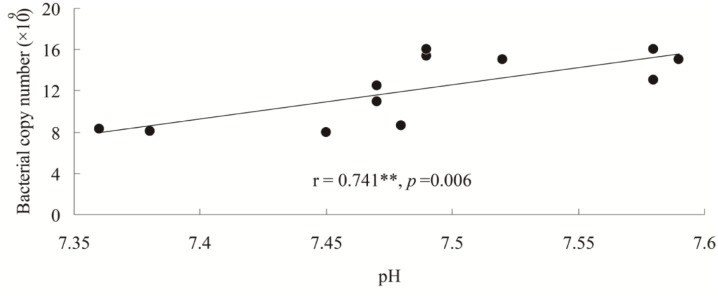
Relationships between the abundance of total bacteria and the soil pH.

qPCR estimates of bacterial population sizes, as determined by 16S rRNA genes, were between 8.32 × 10^9^ and 15.44 × 10^9^ copy numbers g^-1^ dry soil (**Figure 1A**). Fungal ITS rRNA gene copy numbers ranged from 4.59 × 10^7^ to 5.78 × 10^7^ copy numbers g^-1^ dry soil (**Figure 1B**). The population sizes of soil bacteria within Hanhui3T were significantly lower than those of Hanhui3 and Zhonghua11 soils, but higher than in bulk soils. In contrast, the population sizes of soil fungi were not significantly different between Hanhui3T and Hanhui3 (or Zhongua11), but bulk soils had significantly smaller populations. Consequently, the effect of Hanhui3T cultivation on soil bacterial population sizes was greater than the effect on soil fungal population sizes. A pairwise analysis indicated that the abundance of total bacteria was significantly positively correlated (*r* = 0.741, *p* = 0.006) with soil pH (7.41–7.55) (**Figure 2**), but it was not significantly correlated with other soil properties. In contrast, the total abundance of fungi was not significantly correlated to any soil properties.

### Taxonomic Classification and Relative Abundance of Bacteria and Fungi by Illumina MiSeq Sequencing

**FIGURE 3:**
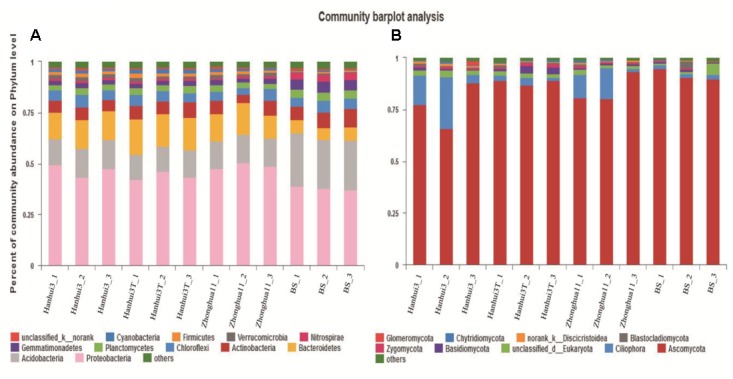
Relative abundance of major bacterial phyla **(A)** and fungal phyla **(B)** in the soil samples. BS indicates bulk soil control.

In total, 171,013 and 333,361 high-quality sequences from 12 samples were obtained by Illumina MiSeq sequencing of bacterial 16S rRNA and fungal 18S rRNA genes, respectively. A total of 36 bacterial phyla, 92 classes, 187 orders, 325 families, and 533 genera were detected in the bacterial dataset. The most abundant bacterial phyla were *Proteobacteria* (44.10%), *Acidobacteria* (16.20%), *Bacteroidetes* (12.60%), *Actinobacteria* (6.70%), and *Chloroflexi* (5.00%), with relative abundances ranging from 36.97 to 50.40%, from 12.48 to 26.20%, from 6.14 to 17.37%, from 4.00 to 9.04% and from 3.33 to 6.14% across all samples, respectively (**Figure 3A**). A total of 26 fungal phyla, 42 classes, 64 orders, 79 families, and 90 genera were detected in the fungal dataset. The most abundant fungal phyla were *Ascomycota* (85.03%), *Ciliophora* (7.50%) and unclassified fungi/Eukaryotes (2.28%), with relative abundances ranging from 65.78 to 94.83%, from 1.51 to 25.27% and from 0.81 to 5.36% across all samples, respectively (**Figure 3B**). There were 2,053 and 219 OTUs that were identified at the 97% similarity level for 16S rRNA and 18S rRNA genes, respectively. Rarefaction curves for all of the soil samples, based on OTUs, reached asymptotes, indicating that the data generated in this study was adequate for the analysis of the bacterial and fungal diversity in the rhizosphere samples collected (Supplementary Figures S1A,B). Moreover, coverage (*C*) based on OTUs, confirmed the adequate sampling of diversity with estimates of 96.39% and 99.87% (Supplementary Table S1) for bacteria and fungi, respectively. The results, taken together, suggest that the sequencing depth used for all 12 of the samples was adequate to detect the total diversity of bacterial and fungal communities.

### Bacterial and Fungal Community Diversity

The alpha-diversity indices represented by the Shannon index *H′*, Simpson index *D*, Richness index *R*, and Chao1 estimate for bacterial communities, were not significantly different between Hanhui3T and Hanhui3 (or Zhonghua11) (**Table 2**). However, the Chao1 estimate of bacterial community richness significantly differed between the rhizosphere and bulk soil communities. Likewise, the Shannon index *H′*, Simpson index *D*, Richness index *R*, and Chao1 estimate for fungal communities were also not significantly different between Hanhui3T and Hanhui3 (or Zhonghua11) (**Table 3**). Notably, the abovementioned diversity index showed significant difference between rhizosphere and bulk soil fungal communities.

**Table 2:** The Shannon–Wiener index *H′*, Simpson index *D*, Richness *R*, and Chao1 of the soil bacterial communities of different rice varieties^a^.

Rice sample	*H′*	*D*	*R*	Chao1
Hanhui3T	6.06 ± 0.07^a^	0.0066 ± 0.0009^a^	1508.33 ± 21.13^a^	1859.82 ± 44.07^a^
Hanhui3	6.01 ± 0.20^a^	0.0082 ± 0.0025^a^	1493.67 ± 184.08^a^	1807.04 ± 117.34^a^
Zhonghua11	5.88 ± 1.00^a^	0.0095 ± 0.0018^a^	1392.67 ± 43.25^a^	1759.45 ± 52.38^a^
BS	5.98 ± 0.07^a^	0.0060 ± 0.0005^a^	1304.67 ± 48.29^a^	1586.90 ± 34.44^b^

^a^*Values represent the averages of three repetitions and the standard deviations. Different lowercase letters in the same column indicate a statistically significant difference at 0.05 level. BS indicates bulk soil control*.

**Table 3:** The Shannon–Wiener index *H′*, Simpson index *D*, Richness *R*, and Chao1 of the soil fungal communities of different rice varieties^a^.

Rice sample	*H′*	*D*	*R*	Chao1
Hanhui3T	2.62 ± 0.04^ab^	0.1451 ± 0.0026^b^	133.33 ± 4.04^a^	146.61 ± 8.15^ab^
Hanhui3	2.71 ± 0.09^a^	0.1285 ± 0.0106^b^	141.33 ± 14.36^a^	150.14 ± 15.36^ab^
Zhonghua11	2.69 ± 0.20^a^	0.1302 ± 0.0306^b^	140.67 ± 4.73^a^	168.22 ± 10.82^a^
BS	2.36 ± 0.17^b^	0.1948 ± 0.0333^a^	111.67 ± 11.55^b^	125.20 ± 7.25^c^

^a^*Values represent the averages of three repetitions and the standard deviations. Different lowercase letters in the same column indicate a statistically significant difference at 0.05 level. BS indicates bulk soil control*.

**FIGURE 4:**
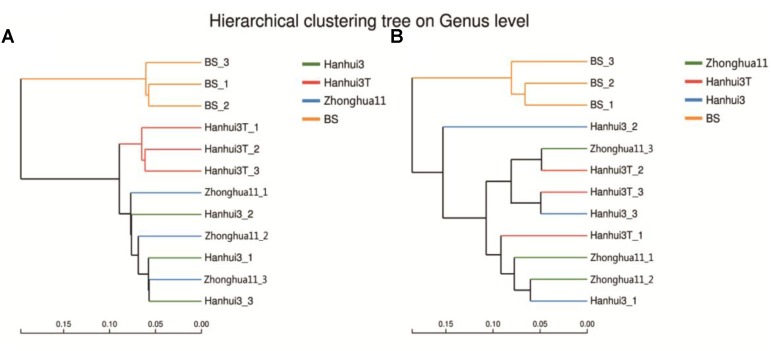
Hierarchical cluster analysis of the bacterial **(A)** and fungal **(B)** communities from different rice varieties rhizosphere and bulk soil based on the Hellinger distances of microbial communities. BS indicates bulk soil control.

Hierarchical cluster analysis of the bacterial communities indicated that the rhizosphere communities from the three rice varieties and bulk soils belonged to two separate groups. This observation suggests an influence of root exudates on bacterial community composition (**Figure 4A**). In addition, the rhizosphere communities from the Hanhui3T and conventional rice variety (Hanhui3 and Zhonghua11) cultivar clustered into two separate groups, indicating that cultivation of insect-resistant and drought-tolerant rice considerably influenced bacterial community composition. Cluster analysis of fungal communities revealed similar clustering patterns between rhizosphere and bulk soil (**Figure 4B**). However, communities from Hanhui3T and Hanhui3 (or Zhonghua11) did not segregate into two distinct groups, suggesting that the cultivation of insect-resistant and drought-tolerant rice did not affect fungal community composition as drastically as that of bacteria. Analysis of similarity (ANOSIM) showed that the difference in bacterial community composition in both Hanhui3 and Zhonghua11 compared to the Hanhui3T was significant (sample statistic (Hanhui3/Hanhui3T) = 0.719, *p* = 0.017; sample statistic (Zhonghua11/Hanhui3T = 0.724, *p* = 0.048), whereas the community composition of Hanhui3 and Zhonghua11 did not differ. However, the ANOSIM assay showed no significant difference among the fungal microbial communities from different soil samples.

### Correlations Between Bacterial and Fungal Community Composition and Soil Properties

**FIGURE 5:**
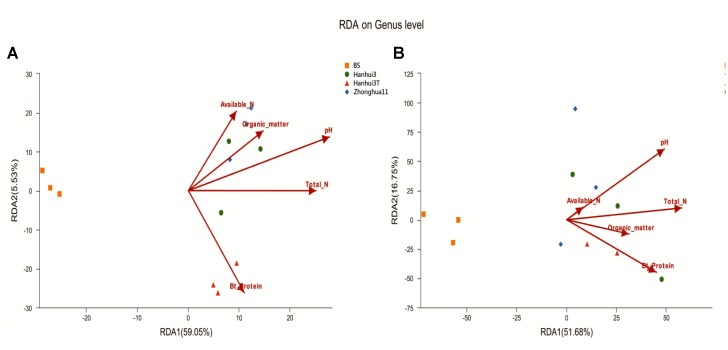
RDA results of the soil properties and bacterial **(A)** and fungal **(B)** community compositions at the genus level. BS indicates bulk soil control. The number on each axis indicates the percentage of the total variation explained.

The relationships between soil properties and bacterial and fungal community compositions, at the genus level, were analyzed by RDA (**Figures 5A,B**). The first two RDA components explained 64.58% of the total variation in bacterial community composition and 68.43% of the total variation in fungal community composition. Soil pH was most correlated with variation in bacterial community composition (*r* = 0.75, *p* = 0.024), followed closely by Bt protein concentrations (*r* = 0.73, *p* = 0.024). However, the other environmental variables were not significantly correlated (*p* > 0.05) to bacterial community variation. In contrast, none of the aforementioned soil variables were significantly correlated (*p* > 0.05) to variation in fungal communities. Spearman’s rank-order correlation was used to assess the response of specific bacterial and fungal genera to soil Bt protein concentrations (Supplementary Tables S2, S3). Thirty-four bacterial genera and seven fungal genera were correlated significantly with soil Bt protein concentrations. The relative abundances of bacterial genera, including *Coxiella*, *Blastopirellula*, *Sorangium*, and *Pseudomonas*, were positively correlated with Bt protein concentrations, while *Gemmatimonas*, *Flavisolibacter*, *Flavitalea*, and others, were negatively correlated with Bt protein concentrations. In addition, the relative abundance of unclassified fungal genera from the *Vampyrellidae*, *Agaricomycetes*, and *RM2-SGM5* groups, in addition to *Neourostylopsis*, were significantly positively correlated with Bt protein concentrations, whereas abundances of unclassified genera from the *Craspedida*, *Malasseziales*, and *Savoryellales* were significantly negatively correlated with Bt protein concentrations.

## Discussion

### Cultivation of Drought-Tolerant and Insect-Resistant Rice Altered Bt Protein Contents and Soil pH

It has been widely reported that Cry proteins can be released into soils in the root exudates of Bt plants during growth (Saxena and Stotzky, 2001; Rui et al., 2005; Knox et al., 2007). In the present study, the content of rhizosphere Bt protein was significantly higher in Hanhui3T soils compared to Hanhui3 and Zhonghua11 soils at the booting stage. Rhizosphere Bt protein concentrations from Hanhui3T were the highest at the booting stage of all of the growth stages, prompting our assessment of the rhizosphere microbial communities at this stage. Notably, we observed significantly lower pH in the Hanhui3T soils compared to Hanhui3 and Zhonghua11, while other soil factors were equivalent. This finding contrasts with a previous study that did not observe significant differences in soil pH between the cultivation of drought-tolerant transgenic rice and the parental rice cultivar (Sohn et al., 2016). The observed decrease in pH could have been caused by degradation of Bt proteins, which may lead to increased availability of amino acids, release of ammonia and subsequent nitrification (Dohrmann et al., 2013; Gundersen and Rasmussen, 2013). Hence, we characterized the proportion of Cry proteins in the total protein content of the rhizosphere, which was determined to be low (Supplementary Material and Methods). Consequently, we speculated that the increase of a small proportion of Cry protein due to genetic modification is unable to alter soil pH significantly, when considering the total protein content in the rhizosphere. Another explanation for the observed pH differences is related to differences in root exudates in the vicinities of roots from the different rice varieties (Somers et al., 2004). Thus, we cultured three rice varieties in International Rice Research Institute (IRRI) solution and found that the total amount of organic acids in root exudates from Hanhui3T was dramatically higher than compared to Hanhui3 and Zhonghua11 (Supplementary Table S4). Thus, the lower pH of rhizosphere soils of Hanhui3T may be related to the clear increase in organic acid abundances in its root exudates when compared to control varieties. Hartmann et al. (2009) found that root exudates containing particular organic acids can modulate local environmental conditions, such as pH, resulting in microbial community variation. However, whether other organic compounds of root exudates could also affect soil pH requires further investigation.

### Effect of Drought-Tolerant and Insect-Resistant Rice Cultivation on Total Bacterial and Fungal Abundances

Cotta et al. (2014) suggested that microbial community abundances should be studied as bioindicators when monitoring for possible impacts of genetically modified plant cultivation. Sohn et al. (2016) did not detect significant differences in microbial community abundances in the soils of drought-tolerant transgenic rice and the parental rice cultivar using plate counts. However, considering that only a small fraction of soil microbial communities are cultured (Hill et al., 2000), we used real-time qPCR to accurately estimate bacterial and fungal abundances. Our results challenged the above conclusion based on the plate counting data. We found the abundance of bacteria in the drought and insect resistant rice rhizosphere was lower than those associated with the drought resistant only rice and conventional rice, although it was still higher than that in the bulk soil. In contrast, total fungal abundance of Hanhui3T was only significantly higher than that of bulk soils, and did not differ from the other two conventional rice varieties. Accordingly, correlation analysis was conducted to quantify the relationships between soil characteristics and microbial community abundances. Among the soil parameters that were tested, only soil pH was positively correlated with bacterial 16S rRNA gene copy numbers, suggesting that pH was the main driver of bacterial abundances. These results are consistent with those of Rousk et al. (2010), who used qPCR and a bar-coded pyrosequencing technique, and found that both the relative abundance and diversity of bacteria were positively correlated to pH, while the relative abundance of fungi was unaffected by pH. However, it should be noted that the components of Bt protein that is released in root exudates could bind to soil humic acids rapidly, resulting in inefficient recovery and quantification of Bt protein concentrations (Crecchio and Stotzky, 1998; Valldor et al., 2015). Nevertheless, our results revealed that the abundance of bacteria and fungi was significantly different between rhizospheres and bulk soils, indicating that rice plants interacted with, and affected rhizosphere microbial communities. Furthermore, Badri and Vivanco (2009) found that the release of root exudates in the rhizosphere supported a highly specific diversity of microbes. Our results in combination with their findings suggested that there is a close co-evolutionary link between plant genotypes and rhizosphere population recruitment.

### Effect of Drought-Tolerant and Insect-Resistant Rice Cultivation on Rhizosphere Bacterial and Fungal Community Diversity and Composition

In contrast to low-throughput sequencing methods, which typically limit analysis to the most dominant phylotypes in microbial communities (Lee et al., 2011; Li et al., 2014), the Illumina MiSeq sequencing approach applied here detected 533 bacterial genera and 90 fungal genera. Consequently, these high-throughput analyses allowed the detection of much less abundant members by a factor of more than two orders of magnitude relative to lower-throughput sequencing methods (Dohrmann et al., 2013). Although soil bacterial community diversity indices (*H′*, *D*, *R*, and Chao1) did not differ significantly between Hanhui3T and Hanhui3 (or Zhonghua11), distinct bacterial community structures were present among these samples. Specifically, Hanhui3 and Zhonghua11 clustered together firstly, suggesting varieties related to the genetic modification had remarkable effect on bacterial community composition. No apparent changes in fungal community composition between drought-tolerant and insect-resistant rice and conventional varieties were found, supporting the hypothesis that bacterial communities are more sensitive to environmental factors than fungal communities (Taylor et al., 2010). Indeed, previous studies have indicated that fungal communities have greater resistance to changes in environmental factors compared to bacterial communities (Kennedy et al., 2004, 2005). Differences in the bacterial community structure between Hanhui3T and Hanhui3 (or Zhonghua11) were hypothesized to result from unintentional changes in root exudate composition or by direct effects resulting from soil chemical parameters on soil microorganisms. RDA indicated that soil pH and Bt protein concentrations were significantly related to variation in bacterial community composition, but that other soil parameters had no significant influences. Although soil pH is commonly regarded as the primary variable responsible for soil bacterial community changes, our results indicated that the impacts of other environmental variables on bacterial community composition varied with soil samples. However, owing to the difficult characterization of rhizosphere organic compounds within root exudates, more studies are required to further evaluate the ecological and co-evolutionary role of root exudates in shaping soil microbial communities. Valldor et al. (2015) demonstrated that the Bt protein of GM crops could be utilized by microorganism as a growth substrate using ^14^C-tracer studies in two agricultural soils. In this study, we also found a total of 34 bacterial genera and 7 fungal genera that were significantly correlated to Bt protein concentrations. Whether these genera were real Bt utilized as growth sources remained unclear. This should be confirmed by future investigation. The other possibility is that bacteria from these genera utilized the dead insects killed by Bt toxins and thus statistically correlated with Bt contents.

## Conclusion

In conclusion, this study represents the first ecological risk assessment of the potential effects of drought-tolerant and insect-resistant rice cultivation on rhizosphere microbial communities. Rhizosphere bacterial abundances and community structures were substantially different between drought-tolerant and insect-resistant rice cultivation and conventional rice variety cultivation at the booting stage. However, these effects were not observed for fungal community composition, indicating that the effects of drought-tolerant and insect-resistant rice cultivation were stronger for bacterial community composition compared to fungi community composition. Moreover, bacterial abundance was positively associated with soil pH, while bacterial community composition structure was primarily controlled by soil pH and Bt protein concentrations.

## Author Contributions

XT and FM designed the experiments. PL performed most of the experiments. AP performed some of the experiments. SY contributed materials and analysis tools. HL analyzed data and discussed the result. PL wrote the manuscript. FM edited the manuscript. XT analyzed the data and edited the manuscript.

## Conflict of Interest Statement

The authors declare that the research was conducted in the absence of any commercial or financial relationships that could be construed as a potential conflict of interest.

## Footnotes

1 http://qiime.org/tutorials/tutorial.html

2 http://rdp.cme.msu.edu/

3 http://www.rproject.org/
