# Supplementary material for: Cultivation of Drought-Tolerant and Insect-Resistant Rice Affects Soil Bacterial, but Not Fungal, Abundances and Community Structures
Source: Front Microbiol. 2018 Jun 29;9:1390. doi: 10.3389/fmicb.2018.01390 (PMC6033987; doi:10.3389/fmicb.2018.01390)

**Supplementary Material and Methods**

***Quantification of total proteins from rhizosphere***

A total of 0.5 mL sterile PBST buffer (137 mM NaCl, 27 mM KCl, 100 mM Na_2_HPO_4_, 10 mM KH_2_PO_4_, 0.5 % Tween-20, pH 7.4), 0.7 g of 0.1 mm zirconia-silica beads (Saint-Gobain ,Shanghai, China), six 2.8 mm ceramic beads (Saint-Gobain ,Shanghai, China) and one 6.3 mm ceramic sphere (MP Biomedicals, Illkirch, France) were added to each rhizosphere sample of 0.5 g into 2.0 mL polypropylene screw-cap tubes. The rhizosphere was macerated by four bead beating steps of 45 s at 6.5 m s^-1^ on a FastPrep-24 system (MP Biomedicals) and centrifuged at 16,000 x *g* and 4 °C for 10 min (Dohrmann et al., 2013). The amount of total protein in the supernatants was determined by using Pierce BCA protein assay (Thermo Scientific).

***Quantification of organic acids released in root exudates from the rice varieties***

A total volume of 100 ml culture solution was collected in triplicate for each rice variety from the all the culture solution in the containers, and concentrated using a Concentrator plus (Eppendorf, Cat. 5305 000.304). The concentrated solution was filtered through a 0.45-μm membrane, freeze-dried, and redissolved in 10 mL of deionized water. The samples were passed through a cation exchanger (Amberlite IR-120PLUS) using Na+ and were then freeze-dried and redissolved in 1 mL of deionized water. This was analyzed by high-performance liquid chromatography (Agilent 1200) using an Agilent SB-C8 (4.6×250 mm, 5 μm) column. The mobile phase consisted of 0.05 M phosphate-buffered saline at pH 2.5 and CH_3_CN, and the flow rate was 1.0 mL min−1. Injection volume was 20 μL, with UV detection at 220 nm. Organic acid standards included formic acid, malic acid, acetic acid, oxalic acid and citric acid.

**Cited references**

Dohrmann AB, Küting M, Jünemann S, Jaenicke S, Schlüter A, Tebbe CC (2013) Importance of rare taxa for bacterial diversity in the rhizosphere of Bt- and conventional maize. ISME J, 7: 37e49.

Yang T Y, Liu G L, Li Y C, Zhu S M, Zou A L, Qi J L, Yang Y H (2012) Rhizosphere microbial communities and organic acids secreted by aluminum-tolerant and aluminum-sensitive soybean in acid soil. Biol Fertil Soils, 48:97-108.

**Supplementary Table S1.** The coverage *C* of the soil bacterial and fungal communities of different rice varieties.

**Supplementary Table S2.** List of the bacterial responsive genera, the abundance of which was significantly correlated with Bt protein content.

**Supplementary Table S3.** List of the fungal responsive genera, the abundance of which was significantly correlated with Bt protein content.

**Supplementary Table S4.** Types and quantities of the secreted organic acids in the culture solution (μg/L) (N = 3).

**Supplementary Figure S1.** Rarefaction curves of Bacterial 16S rRNA (A) and fungal 18S rRNA (B) genes clustered into OTUs at a sequence similarity ≥ 97% (OTU). BS indicates bulk soil control.

Supplementary Table S1.

|  | Hanhui  3T_1 | Hanhui  3T_2 | Hanhui  3T_3 | Hanhui  3_1 | Hanhui  3_2 | Hanhui  3_3 | Zhonghua11_1 | Zhonghua11_2 | Zhonghua11_3 | BS  _1 | BS  _2 | BS  _3 |
| --- | --- | --- | --- | --- | --- | --- | --- | --- | --- | --- | --- | --- |
| Bacterial  community  Fungal  community | 0.9677  0.9996 | 0.9700  0.9992 | 0.9702  0.9987 | 0.9639  0.9994 | 0.9836  0.9996 | 0.9657  0.9992 | 0.9695  0.9992 | 0.9648  0.9991 | 0.9655  0.9991 | 0.9746  0.9991 | 0.9771  0.9995 | 0.9752  0.9993 |

BS indicates bulk soil control.

Supplementary Table S2.

| norank_c__Cyanobacteria | unclassified_f__Oxalobacteraceae | Emticicia |
| --- | --- | --- |
| Flavisolibacter | norank_f__Blastocatellaceae__Subgroup_4 | Gemmatimonas |
| norank_f__PHOS-HE51 | Niastella | norank_f__NS9_marine_group |
| Pseudomonas | Sorangium | norank_f__Moraxellaceae |
| unclassified_c__Alphaproteobacteria | norank_o__Chthonomonadales | unclassified_f__Blastocatellaceae__Subgroup_4 |
| Blastopirellula | Tropicimonas | Coxiella |
| Simiduia | unclassified_f__Xanthomonadaceae | norank_f__A0839 |
| OM60_NOR5__clade | Paracoccus | Candidatus_Aquiluna |
| Zavarzinia | CL500-3 | Flavitalea |
| norank_o__SAR324_clade_Marine_group_B | Amphiplicatus | Methylomicrobium |
| norank_f__JG35-K1-AG5 | norank_f__Coxiellaceae | norank_o__Mollicutes_RF9 |
| norank_o__Myxococcales | norank_f__Unknown_Family_o__Alphaproteobacteria_Incertae_Sedis | |

Supplementary Table S3.

| norank_c__RM2-SGM58 | unclassified_c__Agaricomycetes | unclassified_c__Craspedida |
| --- | --- | --- |
| Neourostylopsis | norank_o__Savoryellales | norank_o__Malasseziales |
| norank_c__Vampyrellidae |  |  |

Supplementary Table S4.

| Varieties | Formic acid | Malic acid | Acetic acid | Oxalic acid | **Citric acid** |
| --- | --- | --- | --- | --- | --- |
| Hanhui3T  Hanhui3  Zhonghua11 | 868±67^a^  187±25^b^  214±32^b^ | 107±15^a^  121±25^a^  97±20^a^ | 128±26^a^  135±23^a^  116±18^a^ | 156±13^a^  58±8^b^  67±11^b^ | 58±12^a^  48±11^a^  63±12^a^ |

^a^ Values represent the averages of three repetitions and the standard deviations.

Different lowercase letters in the same column indicate a statistically significant difference at 0.05 level.

Supplementary Figure S1


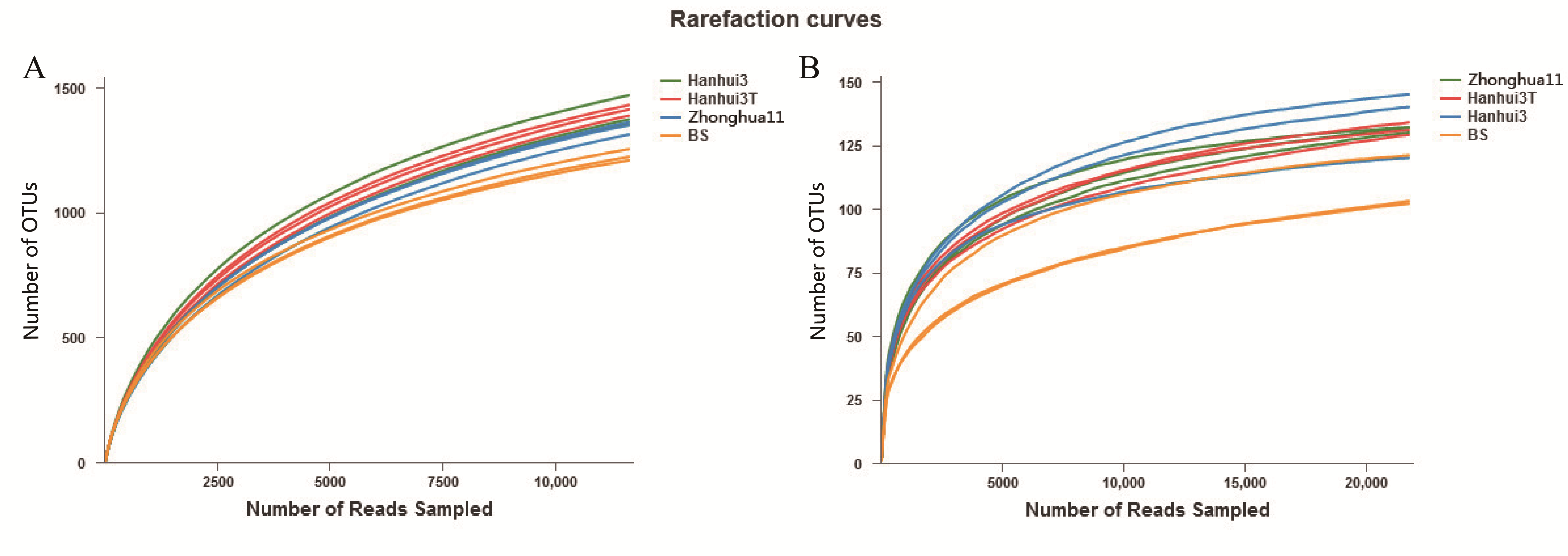

Supplement: Supplementary file 1 [file Data_Sheet_1.DOC]
